# Precautions against Collapse from Anæsthesia

**Published:** 1893-04

**Authors:** 


### Editorial.

### PRECAUTIONS AGAINST COLLAPSE FROM ANÆSTHESIA.

The death of Col. Elliot F. Shepard on March 24th, from inhalation of ether, under the direction of Drs. Charles McBurney and J. W. McLane, is notable, chiefly, from the attending circumstances. It is inferred that there were no antecedent conditions militating against the administration of the anæsthetic in this case; yet he had inhaled the drug but two or three times when the physicians detected dangerous symptoms and stopped the inhalation. At the end of an hour's work with with oxygen, he was restored to partial consciousness and he continued apparently to rally for two hours, when he began rapidly to sink. Notwithstanding the resumption of the oxygen treatment, the patient died in less than a half hour after the unfavorable change. He vomited soon after the commencement of the inhalation of ether, and it then became known, and not previously, that his respirations were not satisfactory, nor his pulse good. This led to the suspicion that some particle of food had lodged in the larynx. But upon examination such proved not to be the case. Even tracheotomy was performed without finding foreign material of any kind in the wind-pipe. A rubber tube was passed down the wind-pipe and into the bronchial tube, and a powerful aspirating syringe was attached to it. Nothing but bloody mucus was detected. It is stated that artificial respiration and every other means which might possibly give relief were resorted to, yet his pulse became steadily more feeble until death occurred. In the opinion of the medical attendants, Col. Shepard died of sudden œdema and congestion of the lungs, following the administration of ether, but primarily due to some cause unknown to them, and perhaps to cerebral anæmia.

It would prove a most ungracious task to undertake to criticize a result of this kind, which is liable to occur with any one, even in the most cautious use of an anæsthetic. Yet, it is very natural that the medical profession should still observe the absence of details in the newspaper accounts as to the process of inhalation adopted, whether by an ordinary cone or some form of inhaler. Nothing is defined as to the mode in which artificial respiration was accomplished, if by the process of Marshall Hall or some other. There is no intimation that electricity was used at any stage or that the head and upper portion of the body were lowered while the feet were elevated, as inculcated by members of the profession high in authority on the treatment of cerebral anæmia.

It will doubtless occur to those who give attention to the measures adopted in this case that the time occupied in passing a tube into the bronchia with aspiration, and in performing tracheotomy, might have been more profitably employed in adopting other means of more efficacy.

On the day this death occurred in New York, I had a patient in the Atlanta Polyclinic who was undergoing preparation for the excision of the left half of the upper Maxillary by the inhalation of the A. C. E. Mixture. Finding that there was undue excitement, I resorted to a small quantity of pure chloroform, and almost as soon as it was inhaled his breathing stopped, and his pulse became nearly imperceptible. I had his arms raised above his head and lowered to his sides at regular intervals and turning his entire body from the back position to the left side alternately, it was my good fortune to get prompt relief.

The use of the A. C. E. Mixture was then resumed and the operation completed under the influence, without further inconvenience. It is not out of place to emphasize my satisfactory experience with the A. C. E. Mixture as an anæsthetic in cases under my care during the past ten years.

I am convinced that its use is less liable to serious results than ether or chloroform uncombined, and would urge its adoption upon my colleagues. I usually make up a six ounce mix ture, using alcohol ℥j, chloroform ℥ii, ether ℥iii, the numbers coming in the order of the letters of the alphabet A. C. E., 1, 2, 3. It was my custom formerly to employ chloroform exclusively, commencing its use soon after it was brought to the attention of the profession by Simpson, and a fatal result from its inhalation has never occurred in my practice nor under my personal observation. But it has often been necessary to interrupt its administration and resort to means of reviving the patient. Sometimes dashing cold water upon the face and giving a few sharp slaps with the hand over the thorax or pressing with short intervals of relaxation over the short ribs has sufficed to restore respiration.

But, again, it has been necessary to employ artificial respiration in various ways, to elevate the lower portion of the subject and even to use the galvanic current to arrest the tendency to vital prostration. I have not ventured upon tne heroic suggestion of acupuncture of the the heart made by Dr. B. A. Watson, and sustained by the results of numerous experiments upon dogs. But in an extreme case of suspended cardiac action, there might be such an impression from a slight puncture as to produce contraction of its muscular fibres and thus set the organ to action again. It would seem proper, therefore, when other expedients has failed, to give this measure a trial.

While considering the resources which may be available for the correction of the deadly collapse from anæsthesia, it is incumbent to advert to the means of prevention which experience indicates to be trustworthy. To evacaute the bowels by a mild laxative and withhold food for six or eight hours previous to administering an anæsthetic is a judicious precaution. A moderate stimulant internally, half an hour in advance, and a hypodermic of morphine and atropia in quantities suited to the state of the patient, fifteen minutes before using the anæsthetic, has been practiced with satisfactory results for many years. Prophylactic measures, however, will not avail in complications of the brain, lungs, heart, or kidney, to which anæsthesia is unsuited.

